# Life in the slowest lane: Feeding allometry lowers metabolic rate scaling in the largest whales

**DOI:** 10.1126/sciadv.adw2232

**Published:** 2025-08-06

**Authors:** Ashley M. Blawas, Simone K. A. Videsen, David E. Cade, John Calambokidis, Ari S. Friedlaender, David W. Johnston, Peter T. Madsen, Jeremy A. Goldbogen

**Affiliations:** ^1^ Hopkins Marine Station, Oceans Department, Stanford University, Pacific Grove, CA, USA.; ^2^ Zoophysiology, Department of Biology, Aarhus University, Aarhus, Denmark.; ^3^ Cascadia Research Collective, Olympia, WA, USA.; ^4^ Oceans Sciences Department, University of California, Santa Cruz, Santa Cruz, CA, USA.; ^5^ Nicholas School of the Environment, Duke University Marine Laboratory, Beaufort, NC, USA.

## Abstract

The hypothesized impacts of whale foraging on ocean productivity are ultimately defined by their metabolic rate, but determining energy expenditure for ocean giants remains challenging. The largest baleen whales use a high-drag lunge-feeding strategy that is hypothesized to come at a high energetic cost, thus requiring exceptional calorie intake. We used biologging tags to measure respiratory rates in foraging rorquals and demonstrate that their field metabolic rates are less than half that predicted by prey consumption estimates and by scaling predictions from smaller marine mammals. The relative cost of rorqual foraging decreases with increasing size as larger whales spend disproportionately longer time filtering prey from engulfed water. By decoupling active swimming and filtration, the largest rorquals forage with limited movement costs. The evolution of lunge feeding confers an energetic advantage that is unique among filter feeders and may have provided an evolutionary pathway to the largest body sizes.

## Teaser

Rorqual whales use “lunge-and-filter” gait to maintain low metabolic rates during foraging despite high-cost feeding strategy.

## INTRODUCTION

Size has long been examined as a fundamental biophysical driver that dictates metabolic allometry in organisms from bacteria to the largest whales. Initially on the basis of empirical data highlighted in the “mouse to elephant curve,” theoretical explanations have failed to support convergence on a singular metabolic scaling exponent among animals (*1*–*4*). This lack of a universal scaling law begs for an understanding of what drives and limits the variability in metabolic scaling across taxa, life histories, and behavioral states (*5*–*9*). The capacity to convert and sustain energy flow is determined by physiological adaptations that define energetic inputs (i.e., foraging, digestion, and assimilation) and outputs (i.e., mechanical work, heat dissipation, tissue growth, and reproduction), thereby revealing how animals evolved to manage energy budgets over time and life histories (*10*). In particular, animals at the extremes of metabolism—the smallest, the largest, the slowest, and the fastest—have great potential to reveal mechanisms for physiological adaptation that enable life at, and beyond, the limits of conventional metabolic models (*11*).

Baleen whales (*Mysticeti*), the most recent and largest radiation of gigantic marine filter feeders, require the highest absolute energy intake across the animal kingdom and are hypothesized to consume an estimated 5 to 30% of their body mass, or ~6 to 16 tons of biomass, per day at peak summer feeding times (*12*). At these caloric requirements, the largest whales are projected to have field metabolic rates (FMRs) that exceed those predicted by FMR scaling from terrestrial mammals (*13*, *14*). Even at FMRs that match predictions for similarly sized terrestrial mammals, like those recently estimated from the oxygen turnover of humpback whales (*Megaptera novaeangliae*) (*15*), the foraging inputs required to support a large body size are extreme. The limited feeding seasons of most mysticetes during the summer months therefore require efficient foraging on their ephemeral and patchily distributed prey to build up lipid stores. Such energy stores, which can reach 35 to 45% of body mass at the end of a foraging season (*16*, *17*), not only fuel their large, fasting bodies during transoceanic migrations to breeding grounds (*18*) but they also must support the substantial costs of gestation and lactation in fasting reproductive females (*19*–*22*).

Rorqual whales (*Balaenopteridae*) are a group of baleen whales—including several of the largest species, like blue whales (*Balaenoptera musculus*) and fin whales (*Balaenoptera physalus*)—that have evolved a specialized lunge filter–feeding strategy. Unlike most large vertebrate filter feeders that use steady swimming to simultaneously engulf and filter prey-laden water at slow speeds (*23*, *24*), rorqual lunge feeding is characterized by a temporally decoupled process of high-speed engulfment of prey-filled water followed by a prolonged filtration phase while gliding (*25*, *26*). By engulfing massive volumes of prey-laden water during dynamic and kinematically complex lunging events (*27*), rorquals capture large quantities of small-bodied prey aggregations, including krill, copepods, and fish, that are retained in the mouth after filtering the engulfed water out through the baleen plates (*25*). Because of an extraordinarily large mouth agape at high speed and the acceleration of engulfed water, lunging has been hypothesized to come at a high energetic cost due to the drag incurred (*28*–*30*). Recently, however, both biomechanical and energetic models have shown that the energetic costs of foraging in lunge-feeding humpback whales are lower than previously thought (*15*). This study suggests that despite a high-drag feeding mechanism, rorquals are able to achieve very low overall foraging costs. Because larger rorquals exhibit relatively larger engulfment capacities, biomechanical models suggest that the cost of lunge feeding should increase allometrically with body size (*30*). However, larger rorquals also exhibit relatively longer filter phases following lunges (*25*), during which locomotion costs should be minimized during glides. Therefore, low foraging costs may not only represent an energetic advantage shared by all rorquals, but such savings could be enhanced at larger body sizes if they scale in parallel with the positive allometry of filtration time.

## RESULTS AND DISCUSSION

### Estimating FMRs using empirical kinematic and physiological data

**Fig. 1.**
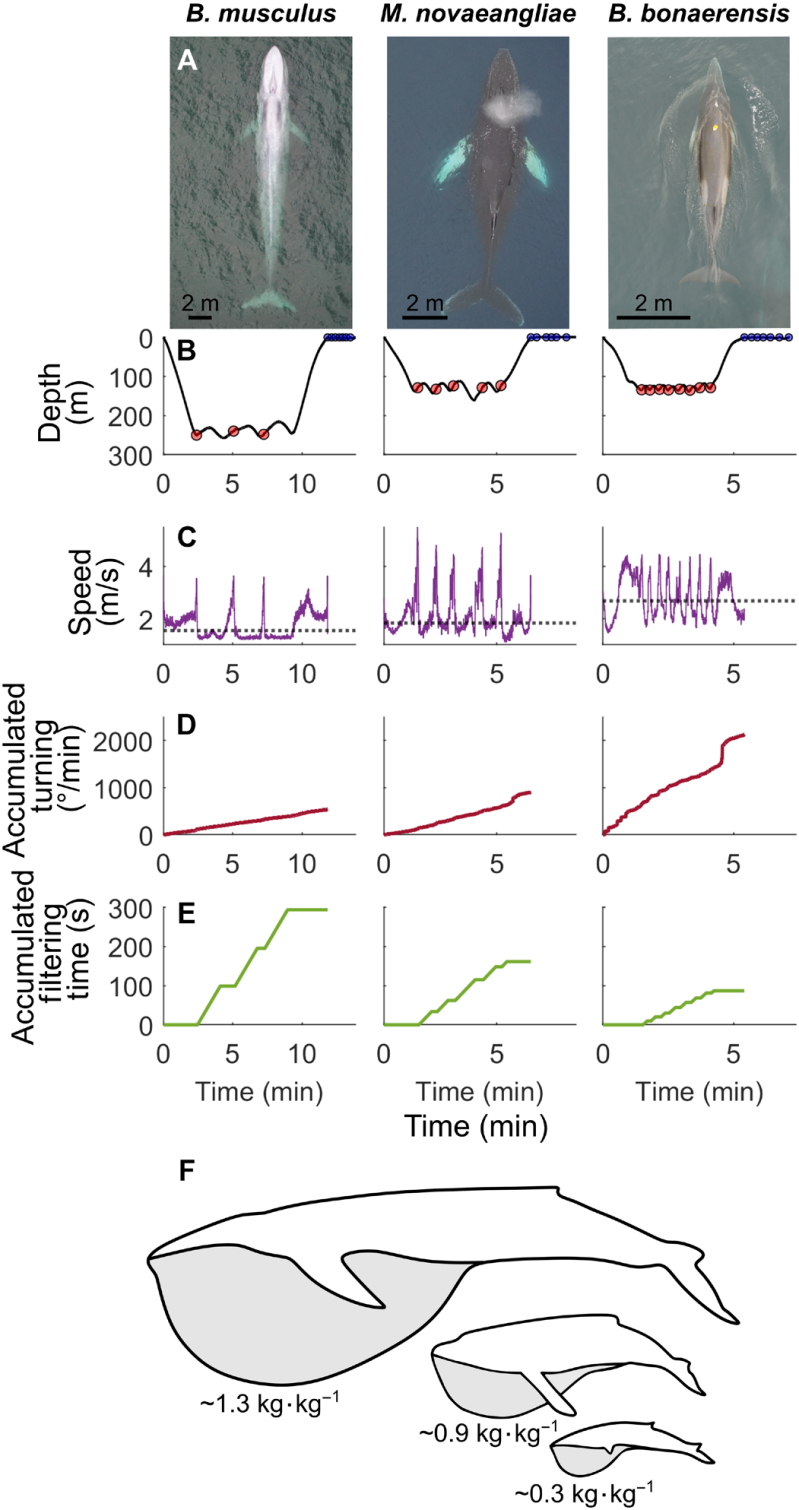
A comparison of feeding dives across three baleen whale species. Relative body lengths (**A**) of blue whales (*B. musculus*), humpback whales (*M. novaeangliae*), and Antarctic minke whales (*B. bonaerensis*) and corresponding (**B**) dive profiles with lunges marked by red circles and breaths for 2 min following each dive marked by blue circles, (**C**) speed profiles with mean speed indicated by a dashed black line, (**D**) accumulated turning, (**E**) accumulated filtering time, and (**F**) diagrams showing increasing mass-specific engulfment capacity with increasing size in rorqual whales. Mass-specific engulfment capacities are estimated using species-specific regressions published in (*113*) using approximate species total lengths of 23, 11, and 6 m for blue, humpback, and Antarctic minke whales, respectively. Image credit: Duke Marine Robotics and Remote Sensing, Duke University.

In this study, we ask whether the high-drag lunge-feeding strategy of the largest baleen whales requires commensurate metabolic rates, as suggested by prey-intake estimates and published scaling relationships (*12*, *14*), or whether the allometry of lunge filter feeding confers energetic savings (*15*). Calculations of rorqual energy expenditure have often relied on metabolic rate scaling extrapolations from smaller animals or hydromechanical models of lunge feeding, both of which may not reflect actual energetic expenditures at these extreme organismal scales. Methods incorporating empirical respiration rates have allowed for field estimates of metabolic expenditure (*31*, *32*) but have not previously accounted for variation in other respiratory parameters (*33*). Recently, respiratory rates, combined with probability distributions of tidal volume and oxygen extraction fractions, have been used to estimate FMR of foraging humpback whales (*15*). Here, we use this methodology to investigate how FMR scales across several baleen whales with body masses that range three orders of magnitude using biologging tags to quantify body kinematics and respiratory rates across the full body size range of rorqual whales, from 4.7-m minke whales (*Balaenoptera bonaerensis*) to 25.2-m blue whales (Fig. 1, A and B). We calculated the scaling of FMR during feeding and nonfeeding periods by modeling oxygen consumption as a function of tag-measured respiration rates and drone-based body length measurements. We then combined detected respirations with estimated tidal volumes and fractional oxygen uptake to determine energy turnover on a per-breath basis and calculated daily FMR (*15*). We also determined the relationship between body mass and daily FMR and demonstrate how metabolic scope varies at the largest animal body sizes on the foraging grounds.

### Energetic savings of the “lunge-and-filter” gait

Mass-specific engulfment capacity increases with increasing body length more rapidly than baleen filtering area (i.e., relatively bigger volumes of water must be pushed through relatively smaller baleen areas). This results in disproportionally longer filter times in larger rorquals, from 6 s for filtering 550 liters of water in the smallest minke whales up to 80 s for filtering 200,000 liters in the largest blue whales (*25*, *34*). The filtering phase of a lunge-feeding event is stereotyped by unpowered gliding (*25*, *26*), a locomotor gait that has been shown to reduce energetic costs in other marine mammals (*35*). Because mass-specific engulfment capacity increases allometrically with body size, gliding gaits during filtration increasingly dominate the lunge cycle of larger rorqual foraging, leading to lower average speeds (Fig. 1C and fig. S1). Moreover, by lunging at lower rates, the largest whales also limit accumulated turning during feeding (Fig. 1D) (*36*, *37*), affording species like blue whales, which lunge sparingly, an additional potential mechanism for increased energetic savings given the costly nature of maneuvering (*38*, *39*). The allometry of filter feeding morphology and performance therefore allows the largest whales to spend most of their foraging dives in a predominantly gliding locomotor state (Fig. 1E). The ability to forage while not actively swimming represents a unique feature to rorqual life at extreme body sizes and high Reynolds numbers, which is not observed in other large filter-feeding taxa including basking sharks, whale sharks, and balaenid whales [i.e., right whales (*Eubalaena*) and bowhead whales (*Balaena mysticetus*)].

**Fig. 2.**
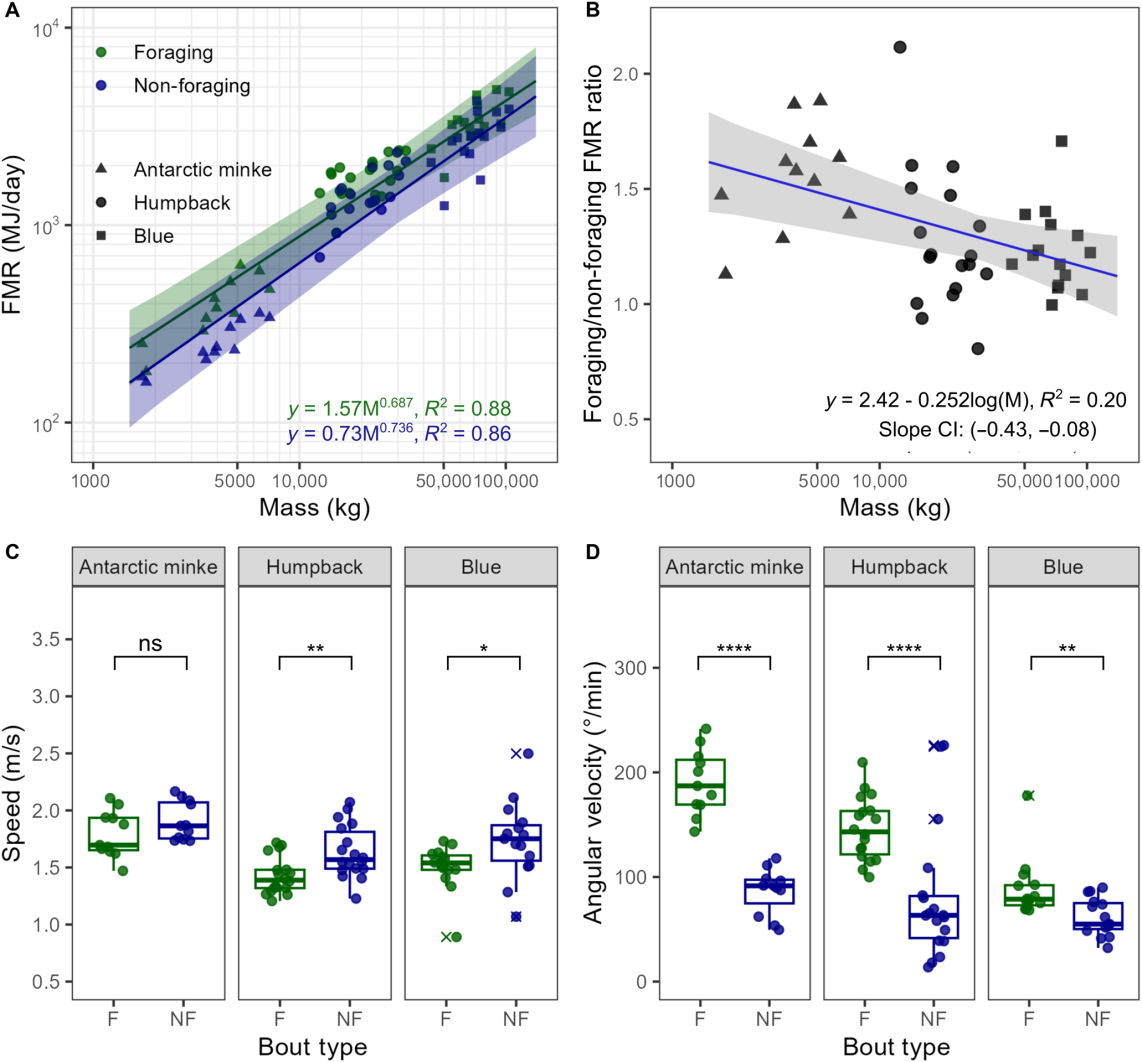
Metabolic scope of three baleen whale species. FMR scaling during foraging (F) and non-foraging (NF) bouts (**A**), (**B**) the ratio of FMR during foraging and non-foraging bouts, (**C**) mean speed during foraging and non-foraging bouts, and (**D**) mean angular velocity during foraging and non-foraging bouts. Each point represents a tagged individual, and outliers of the distribution are designated with x’s. Asterisks indicate the significance of a Wilcoxon rank sum test.

Our measured respiration rates and calculations of FMR demonstrate that although energetic expenditure is between 19.6 and 60.4% higher during foraging compared to nonfforaging periods (Fig. 2A), this metabolic scope is reduced at the largest sizes such that the added cost of feeding for a blue whale is minimal (Fig. 2B). The data suggest that an 80-ton blue whale can reduce its relative foraging costs by an estimated 25% compared to a 5-ton Antarctic minke whale. This difference is attributed to blue whales maintaining slower mean speeds and minimizing fine-scale maneuvering during foraging bouts (Fig. 1, C and D). The convergence of feeding and nonfeeding FMRs may reveal a previously unknown mechanism for energetic savings in rorqual whales and demonstrates an additional advantage of extreme body size in this lineage. Increased feeding efficiency and the energy surplus it can generate supports theoretical models predicting the evolution of large body size under a series of energetic trade-offs (*11*, *40*). If the energetic cost of feeding is reduced at larger body sizes, as shown by our results, previous estimates for the scaling of foraging energetic efficiency (*41*) may scale more favorably (i.e., steeply) with body size for the same level of prey availability. Consequently, this may further ease maximum body size constraints in rorquals. In this sense, the morphological adaptations associated with filter-feeding generate biomechanical cost savings that makes the lunge-feeding strategy more energetically efficient than previously predicted for the largest rorquals (*41*). The lunge-and-filter analog to the “burst-and-coast” and “stroke-and-glide” locomotory strategies used by fish (*42*) and other marine mammals (*35*, *43*–*45*) reinforces the energetic advantage of intermittent swimming styles. By decoupling engulfment from filtering, lunge feeders can achieve an overall efficient locomotory strategy that compensates for the high, but short power output of the engulfment phase (*34*).

### Prey consumption’s hidden costs

FMRs reflect the sum of basal metabolic rate (BMR), specific dynamic action (SDA; i.e., the cost of food processing and absorption), and the cost of locomotion (COL). The ability to decouple feeding from locomotion directly reduces COL, thus lowering overall FMR in larger baleen whales. While it is unlikely that BMR is changing on the timescales studied here, SDA could play a role in the FMRs observed. Yet, little is known about the magnitude and timing of SDA in most free-ranging marine mammals, including whether food processing occurs during dives characterized by circulatory changes via the dive response that minimize blood flow to the digestive organs (*46*). In smaller marine mammals, SDA has been measured at 5 to 15% of energy intake with intra-species variability depending on prey type and meal size (*47*–*49*). If large-bodied whales incur a portion of the metabolic costs of food processing, absorption, and protein synthesis while foraging, then our foraging bout FMR estimates could overestimate foraging costs (i.e., an increase in FMR measured during foraging relative to non-foraging). Observation of lipemic blood and sustained hepatic blood flow in diving Weddell seals suggests the possibility of sustained digestion during deep foraging dives (*50*). Blue whales demonstrate exercise-induced tachycardia during lunge feeding (*51*), which suggests that concomitant increases in regional blood flow occur during foraging events (*52*). Such pulses of perfusion could support digestion during otherwise minimal perfusion of splanchnic organs during diving, as observed in the Weddell seal (*53*), in addition to presumed normal digestive function during surface intervals. Like other marine mammals, rorquals may partially delay digestion to resting surface intervals rather than during dives, suggesting that digestion costs may be distributed unequally between dive and surface time (*54*). In sum, it seems possible that large rorquals may be able to digest prey relatively continuously during foraging bouts and during the succeeding non-foraging bouts as needed, leading to a potential overestimate of energetic costs due to foraging reflected in reported FMRs.

Another potential driver of increased FMR in feeding in rorquals is the thermoregulatory cost of consuming cold prey or the “ice cream effect” (*55*). Lunge feeding requires rorquals to engulf and, for the duration of filtering, retain large volumes of water that may be 20° to 40°C below body temperature. Evidence in gray whales (*56*, *57*) and bowhead whales (*58*) suggests that extensive counter-current heat exchangers create an efficient thermal gradient that functions to dramatically limit heat loss through the tongue (*56*). However, these counter-current heat exchangers may be bypassed via shunting so that the oral cavity could also serve as important thermal windows for dumping heat in these large-bodied whales (*58*–*60*). Heat transfer during feeding can occur not just to water but also to the cold prey consumed. Each engulfment ends with swallowing a large quantity of water-temperature prey that is transported into the well-insulated core of the body for digestion. As rorquals’ prey has high water content, and therefore high specific heat capacities, substantial energy is required to heat up the ingested prey to body temperature (*55*). This thermoregulatory cost has the potential to increase FMR considerably in feeding marine endotherms. For example, an Adelie penguin is expected to spend up to 8% of its daily energy expenditure warming prey (*55*, *61*). However, very large whales such as rorquals have much more favorable surface to volume ratios (*62*) compared to small marine endotherms like penguins. Thus, they likely never face the challenge of staying warm but often need to actively work to stay cool. Accordingly, in rorquals, it is possible that cold prey acts as a heat sink to offset the heat produced by the skeletal muscle during lunge acceleration (*63*) that would otherwise have to be dumped to the environment. In this case, no new heat production would be needed to warm prey; thus, it is, in our view, unclear whether the ice cream effect contributes to increased FMR during feeding. If the ice cream effect does contribute to energetic costs during foraging period, this only reinforces our assessment of cheap foraging in rorquals as it implies that the biomechanical costs of feeding are even lower than our estimates.

### Ecological implications of low foraging costs

Our FMR calculations for baleen whales on their foraging grounds are less than half those previously estimated from prey consumption rates (*12*) but on par with scaling predictions from eutherian mammals (*64*). Reduced FMRs compared to these high estimates (*12*) ease the requirement for rorquals to forage only on high-density prey patches and support their use of relatively low-density prey patches as well (*15*, *65*, *66*). Whales cannot survive on the mean concentration of prey on ocean basin scales and thus must navigate between concentrated prey patches to feed (*67*). Optimizing energetic efficiency at both dense and sparse prey patches (*66*) not only eases the requirements for foraging opportunities but also that for locating and navigating between prey patches across large spatial scales. At low mass-specific FMRs, energy accumulation and fasting ability increase (*22*), thereby relaxing the requirement of consistent ocean conditions that generate dense prey aggregations (*15*). Similarly, relaxed requirements for patch quality support the observation of repetitive lunging within a prey patch even as individual prey items are depleted (*68*). Thus, inexpensive foraging allows the planet’s largest animals to target the biggest food resource on the planet more widely despite its ephemeral nature (*69*, *70*).

The fundamental size-driven constraints on structure and function dictate that the largest rorquals should exhibit an energetic buffer against environmental changes that affect prey abundance and distribution (*71*). The combined allometry of lipid stores (i.e., blubber) and FMR implies that larger animals will be able to fast longer—up to 8 times longer for an animal 1000 times heavier (*69*)—making them more resilient to limited prey resources. How changing oceans will shift the abundance and distribution of prey is unclear, but low foraging FMRs afford the largest whales a buffer to the fluctuating conditions of future oceans. Blue whales use long-term memory to track upwelling-driven productivity and foraging hot spots (*72*), a hallmark of the baleen whale food web known as “wind to whales” (*67*). Thus, the oceanographic changes related to upwelling will be critical factors that influence large whales’ ability to successfully forage (*73*). The extreme body sizes, greater than 15-m body length (i.e., *B. physalus* and *B. musculus*) and more than 50-ton body mass (*74*), observed among the largest rorqual species are a recent evolutionary phenomenon that coincided with intensified ocean upwelling since the Plio-Pleistocene (*70*). If prey is not limited, then the calculated rates of maximal body size evolution among mammals (*75*) suggest the possibility that some rorqual whale lineages may be poised to evolve even larger body sizes in the future.

### Evolutionary consequences of low foraging costs

**Fig. 3.**
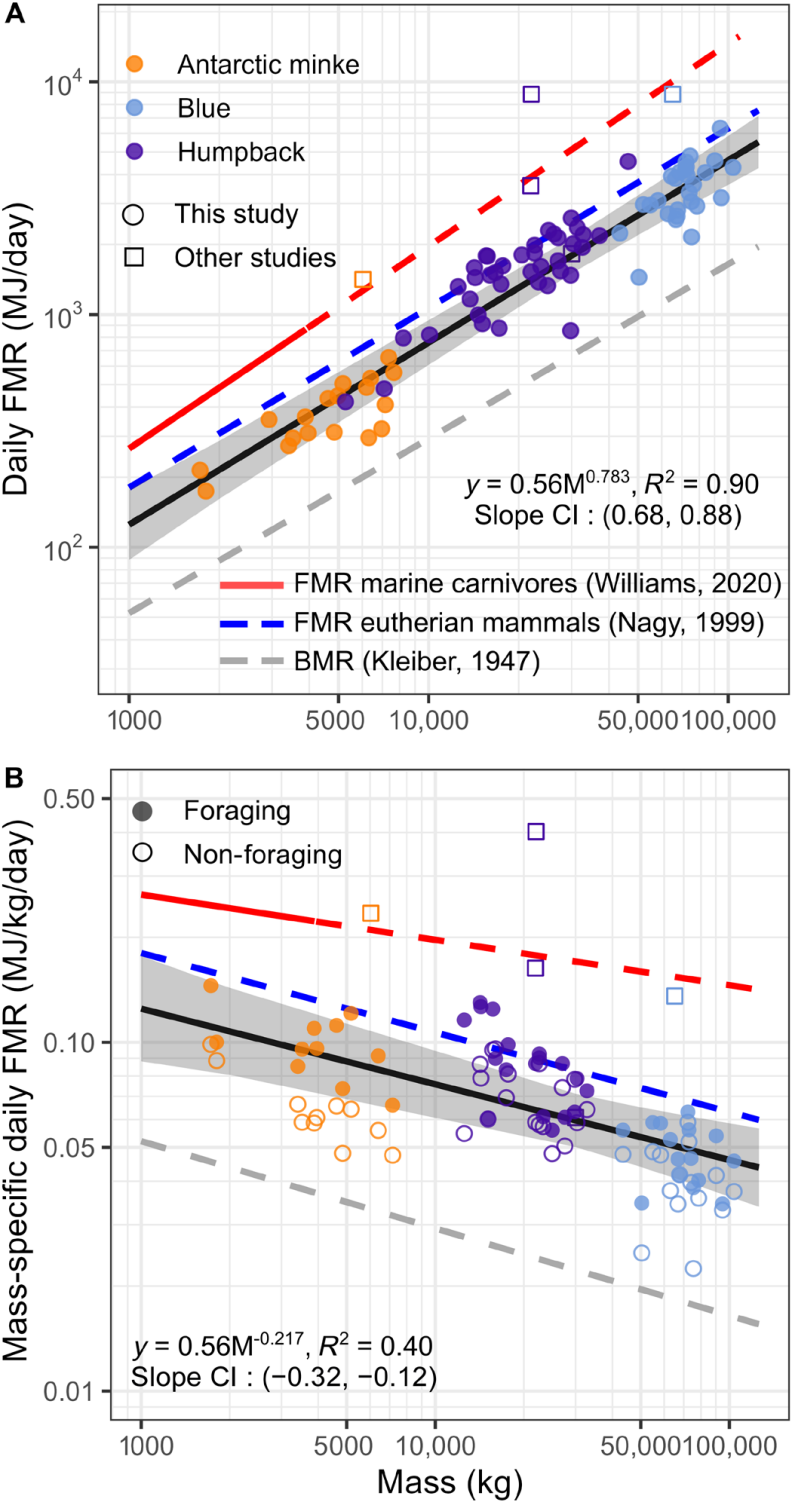
Scaling of FMRs and mass-specific FMRs of baleen whales on the foraging grounds. Each point represents the body mass and FMR of a tagged individual. Circles indicate data from this study, and squares indicate data from other studies (*12*, *15*). The solid black line in both plots represents the fixed-effect models represented by the provided regression equation of FMRs (**A**) and mass-specific FMRs (**B**) across all tag records (*n* = 83) with the marginal coefficient of determination (*R*^2^) value and 95% confidence intervals (95% CIs) for the regression slope. CIs (95%) are shaded in gray. The red, blue, and dashed gray lines represent previously published regressions for metabolic rate scaling relationships (*14*, *64*, *114*). For these previously published regressions, a solid line indicates the body mass range that was included in the data collected and a dashed line indicates the regression plotted into an extrapolated body mass range. In (A), each whale (*n* = 83) is represented by one point, which includes foraging and non-foraging periods. In (B), only whales included in the bout analysis are plotted as points with each whale (*n* = 44) represented by two points, one for foraging bout FMR (closed circles) and one for non-foraging bout FMR (open circles).

Our results show that large baleen whales exhibit FMRs on the foraging grounds that are less than half that predicted from extrapolating metabolic scaling in smaller marine mammals (i.e., odontocetes and pinnipeds) (Fig. 3, A and B) (*14*, *76*). Although variance around allometric predictions at the extremes of body mass should be expected, the disagreement between the FMRs of baleen whales and previous scaling relationships for small marine mammals, which demonstrate FMRs elevated beyond eutherian mammalian scaling, suggests that the drivers of metabolic rate differ across marine mammal groups. This difference could be driven largely by the lack of thermoregulatory costs at favorable surface area to volume ratios at this scale (*77*) and low costs of transport at high Reynolds numbers. In addition, as scaling exponents within a clade may result from selection to optimize the needs of that clade’s life history strategy (*9*), it could reflect differences in the life history traits of rorquals compared to other taxa. Metabolic rates in animals are expected to be consistent with “pace of life” characteristics (*78*) like female age at sexual maturity, longevity, interbirth interval, weaning duration, gestation period, and litter size (*79*–*81*). Rorquals exhibit an extremely fast pace of life for their body size with low mass-specific generation times, high production rates (*5*), and reduced gestation and lactation times (*82*). Yet, our results demonstrating lower FMRs of foraging baleen whales compared to other placental mammals, combined with evidence for even lower FMRs on the breeding grounds (*83*), suggests that baleen whales are able to energetically compensate for their fast pace of life (*84*, *85*). Perhaps the combination of low metabolic costs and efficient feeding on large prey patches enables the allocation of more energy per unit time for reproduction. In this case, the energetic savings due to an increasing ability to feed without actively swimming (i.e., the savings due to a higher proportion of a lunge-feeding event spent doing no-cost filtering) at larger body sizes may contribute directly to a larger excess of daily energy available to allocate towards reproduction and growth.

It should also be considered that unlike smaller, income-breeding marine mammals that must physiologically support the possibility of reproduction year-round, capital-breeding baleen whales are limited to discrete reproductive seasons. Therefore, it is possible that decreased expenditure toward maintaining reproductive viability, lactation, and weaning while off the breeding grounds helps maintain low FMRs while on the foraging grounds. The observation of lower metabolic rates during the breeding/resting season (*83*) could also reflect an adaptive response to energy restriction as fat stores are reduced during lactation (*86*). This metabolic plasticity would support a lower metabolic overhead during nonreproductive seasons and support a model of constrained energy expenditure in the largest whales, as observed in humans and other animals (*78*, *87*). The plasticity of metabolic investment may therefore be a crucial adaptation that affords baleen whales a life in the slowest lane during foraging while maintaining a fast pace of life for reproduction. In addition to large size and long lifespans, the ability to achieve such energetic trade-offs may also underpin an additional superlative of cetaceans by powering the longest migrations observed among animals (*88*). For the success of future whale populations, low energetic requirements coupled with the most efficient feeding mechanism will enhance large whales’ resilience for coping with anthropogenic threats (*89*) and intensifying environmental change (*90*, *91*).

## MATERIALS AND METHODS

We determined respiration rates and body size of 83 baleen whales (27 blue whales, 38 humpback whales, and 18 Antarctic minke whales) on their foraging grounds (table S1) (*92*, *93*). Tags collected 950 hours of data and recorded 59,454 breaths. Our analysis of foraging and non-foraging bouts included 15 blue whales, 18 humpback whales, and 11 Antarctic minke whales for a total of 44 individuals. These whales were in foraging bouts for a total of 460 hours and in non-foraging bouts for 379 hours. Individuals took a total of 32,878 breaths within foraging bouts and 19,683 breaths during non-foraging bouts (table S2).

### Tag data

Tag data used in this study were collected by short-duration suction-cup attached tags deployed between 2015 and 2023 from baleen whales on their foraging grounds. Customized Animal Tracking Solutions (CATS) tags recorded pressure at 400 Hz, temperature, light, and GPS at 10 Hz and kinematics using a three-axis accelerometer, three-axis gyroscope, and three-axis magnetometer sampling at 400, 50, and 50 Hz, respectively. CATS tags also contained high-resolution video cameras and a single hydrophone, which collected video at either 25 or 30 fps at a resolution of 1280 pixel by 720 pixel or 1920 pixel by 1080 pixel with paired audio at 22.5 kHz with 16-bit resolution.

All tags were deployed using standard methods for remote attachment of suction-cup sensor packages including placement onto the dorsal surface of the animal using a carbon fiber pole from a small rigid hulled inflatable boat (<10 m). Four suction cups attached the tag to the animal, which detached following suction failure, commonly due to conspecific contact or rapid maneuvering, or after a predetermined release duration had been met. After detaching from the animal tags floated at the surface and were retrieved using VHF radio tracking. Tagging was permitted by the National Marine Fisheries Service (NMFS #16111, 14809, 19116, 21678, 20430, and 23095) and approved by the Institutional Animal Care and Use Committee of Stanford University (IACUC #30123), Cascadia Research (AUP-6), and UC Santa Cruz (Friea2306dn).

To reduce the effect of tagging on our analyses, the first hour of data following tag deployment was removed from the dataset. In post-processing steps, the data were decimated to a sampling rate of 10 Hz and custom MATLAB tools were used to determine the animal’s pitch, roll, and heading from accelerometer and magnetometer data (*94*). Pressure was used to determine animal depth after correcting for temperature fluctuations at the pressure sensor. Speed of the tagged individual was determined from the magnitude of the accelerometer jiggle, which rely on in vitro calibrations made in a flow tank as well as in situ calibrations against orientation-corrected depth rate (*95*).

### Body size

Lengths of tagged individuals were estimated using unoccupied aircraft systems (UAS). Several different UAS platforms (quadcopters: DJI Phantom 4, DJI Phantom 4 adv; hexacopters: LemHex 44, Freefly Alta 6) were used to obtain nadir photographs of tagged individuals while they were at the surface. All aircraft had a barometric altimeter, and the hexacopters were also fitted with an aftermarket laser altimeter unit (LightWare SF11-C LIDAR). Quadcopters were flown above 50 m to minimize error of the barometric altimeter, and calibration against the known vessel length was used to validate barometric altimeter readings (*96*). The total length (TL) of the imaged whales was measured in pixels from the tip of the rostrum to the fluke notch. Given the known sensor dimensions of each platform’s camera, the camera’s focal length, and UAS altitude at the time the photo was taken, the body length of an individual in pixels was converted to meters using the open-source photogrammetry software MorphoMetriX (*97*). To estimate body mass (*M*_b_) from TL, we used previously established correlations between length and fluid-loss–adjusted mass for baleen whales (*74*).

### Respiration rate

Respiration events were detected in two ways using depth information and movement data during surfacings. Typically, baleen whales take a single breath per brief surfacing (*98*), allowing breathing events to be identified from short excursions (<10 s) to the surface when roll remained within 45° of the surface plane. During some surfacings, when depth remained within 1 m of the surface for >10 s, it was possible that the animal took multiple breaths and therefore a secondary signal was required to identify these breathing events. As whales use rapid flow rates during breathing, the movement of the musculature surrounding the thoracic cavity and rapid air flow at the blowhole result in short increases in measured acceleration on the tag that can be distinguished from background changes in low-frequency motion. Thus, breaths during surfacings >10 s were determined to occur where peaks in the Shannon entropy (SE) of differentiated acceleration signals were identified. All detected breaths were manually inspected to verify co-occurrence of required conditions in depth, roll, and acceleration (*99*). In cases where detection of breaths from peaks in acceleration signals were difficult to distinguish from baseline movement, likely either due to high sea state causing splashing on the tag or tag placement being too far from the thoracic cavity/blowhole, tags were dropped from the analysis. Overall breathing rates were determined as the number of breaths during a tag deployment divided by the tag deployment duration. For foraging and non-foraging bouts, breathing rates were determined as the number of breaths during that bout type divided by the total time spent in that bout type.

### Foraging bouts

Lunge-feeding events for tagged rorquals had been determined previously which, in brief, were identified by identifying stereotypic kinematic signatures indicative of the various phases of a lunge-feeding event (i.e. acceleration, engulfment, and filtering) (*27*). Foraging bouts were defined according to analyses of post-foraging dive surface intervals on a per-species basis conducted in Cade *et al.*, 2021 and Cade *et al.*, 2023 (*92*, *100*). Distributions of surface intervals following foraging dives (i.e., those which included at least one lunge) were fit with Gaussian curves, and the mean value (μ) of the fitted distribution +3 SD in the bulk of the data was taken to be the threshold for defining foraging bouts (*92*, *100*). If the interval between two foraging dives was greater than this value, these were considered to have occurred during separate foraging bouts. In this study, we used previously calculated foraging bout thresholds for Antarctic minke whales (6.0 min), krill-feeding humpback whales (5.5 min), and blue whales (5.5 min) and a newly derived threshold for fish-feeding humpback whales (5.6 min, fig. S2) to define periods of feeding (i.e., time within a foraging bout) and nonfeeding (i.e., time not within a foraging bout) (*92*, *100*). Only bouts >1 hour in duration were retained in the analysis. To address the possibility of a recovery period between foraging and non-foraging bouts, we considered four possible cases for excluding such a transition from the start of non-foraging bouts. Those scenarios included a 1-hour transition, 30-min transition, 15-min transition, and no-transition period. There was no difference in nonfeeding FMRs with transition durations (fig. S3); therefore, we chose to use no-transition period to maximize the number of included data points.

To determine activity during foraging and non-foraging bouts, we determined average speed and average heading velocity for all bouts. Bout speed was calculated by integrating speed for depths >2 m for the entire duration of the bout and dividing the accumulated distance traveled by the bout duration. Bout angular velocity was calculated by integrating the change in heading to determine radians swept within a bout and dividing this by the bout duration.

### Field metabolic rates

Empirically measured respiration rates were combined with tidal volume and oxygen extraction estimates in a Monte Carlo model to estimate FMRs as described in Videsen *et al.*, (*15*). The FMRs reported are the median values of the output distribution. In brief, FMR was calculated by multiplying the number of breaths in a deployment or bout (*N*_breaths_) by the calculated oxygen uptake per breath (*VO*_2 breath_) and converting to kilojoule using the conversion factor 20.08 kJ/liter O_2_ (Eq. 1)

$${\mathrm{FMR}}_{\text{Deployment}\;}=N_{{\text{breaths}}_{\text{Deployment}}}\cdot VO_{2_{\text{breath}}}\cdot 20.08\;\frac{\mathrm{kJ}}{L\;O_{2}}$$ (1)

Respirations were determined empirically, as described above. To determine *V*O_2 breath_, it was necessary to estimate the tidal volume of each breath (*V*_T_) as well as the oxygen extraction coefficient (*E*O_2_), which are multiplied together and then scaled by 0.2095, the fraction of O_2_ present in ambient air (Eq. 2)

$$VO_{2_{\text{breath}}}=V_{T}\cdot EO_{2}\cdot 0.2095$$ (2)

To calculate *V*_T_, total lung capacity (TLC) was estimated from the body mass of each individual using a previously published allometric scaling equation of lung volume from harbor porpoise to fin whale (*101*). Vital capacity (*V*_C_) was assumed to be 85% of TLC (*102*–*106*), and *V*_T_ was assumed to be 60% of *V*_C_ based on respirometry measurements from smaller cetaceans and baleen whale calves, respectively. To account for variability in *V*_T_, the Monte Carlo model sampled from a Gaussian distribution of *V*_T_ values with μ_VT_ = 0.6 ·*V*_C_ and *SD*_VT_ = 0.15 · μ_VT_ (i.e., 0.09 · *V*_C_). To understand the effects of varying *V*_T_ on our estimates of FMR, we reran our model and set *V*_T_ to be a constant high (μ_VT_ = 0.95·V_C_) or low (μ_VT_ = 0.15·V_C_) value (fig. S4). Ninety-five % is the reported high value for *V*_T_s from killer whales (*107*, *108*) and 15% is the low reported value for *V*_T_s from gray whale calves (*109*) of measured *V*_T_s in cetaceans in the literature.

*E*O_2_ is estimated by making several founded assumptions about cetacean gas exchange and the alveolar gas equations. Firstly, it is assumed that *E*O_2_ stabilizes within the time period of the respiration data that is being analyzed. By assuming that dead space is trivial because of the large tidal volumes of cetaceans, *E*O_2_ can be directly estimated from the arterial partial pressure of CO_2_ (P_a_CO_2_). Because P_a_CO_2_ is stable across mammals, it was determined that *E*O_2_ should occupy the range from 0.24 to 0.45 (*110*). Thus, we supplied the Monte Carlo model with a Gaussian distribution of *E*O_2_ values that correspond to this range with mean = 0.35 and SD = 0.03 (*110*).

### Statistical analysis

All statistical tests were performed in RStudio (v.4.1.0) (*111*). To account for repeated measures within a species, all linear relationships were assessed using mixed effects models where the slopes were allowed to vary by species. Mass was used as the predictor variable in all models, and FMR, speed, and turning were the modeled outcome variables. To account for the nonlinear allometry of all predictor variables, mass and all predictors were log_10_-transformed. The scaling of foraging and nonforaging FMR were compared using a two-way analysis of variance (ANOVA) where the interaction of log_10_-transformed mass and feeding status (feeding or nonfeeding) was used to determine whether the slopes of the two models differed, and the null model with feeding status alone was used to determine differences in model intercepts. Assumptions of linearity, homoscedasticity, and normality of residuals were determined to be met by visualizing model residuals. Where coefficient of determination (*R*^2^) values are reported for mixed-effects models, only the marginal *R*^2^ value is reported to indicate the variance described by the fixed effect only. Marginal *R*^2^ values were determined using Nakagawa’s *R*^2^ method for mixed effects models (*112*). Wilcoxon rank sum tests were used to compare speed and angular velocity distributions between species during foraging and non-foraging bouts. Significance was determined using α = 0.05. Data are presented as the means ± SD, unless otherwise stated.

## Supplementary Materials

### This PDF file includes:

Figs. S1 to S4 Tables S1 and S2 References
