# Supplementary material for: Life in the slowest lane: Feeding allometry lowers metabolic rate scaling in the largest whales
Source: Sci Adv. 2025 Aug 6;11(32):eadw2232. doi: 10.1126/sciadv.adw2232 (PMC12327476; doi:10.1126/sciadv.adw2232)
Supplement: Supplementary file 1 — Figs. S1 to S4 Tables S1 and S2 References [file sciadv.adw2232_sm.pdf]

Science Advances
AAAS

# Supplementary Materials for

## Life in the slowest lane: Feeding allometry lowers metabolic rate scaling in the largest whales

Ashley M. Blawas *et al.*

Corresponding author: Ashley M. Blawas, ashleyblawas@stanford.edu

*Sci. Adv.* **11**, eadw2232 (2025)
DOI: 10.1126/sciadv.adw2232

**This PDF file includes:**

Figs. S1 to S4
Tables S1 and S2
References

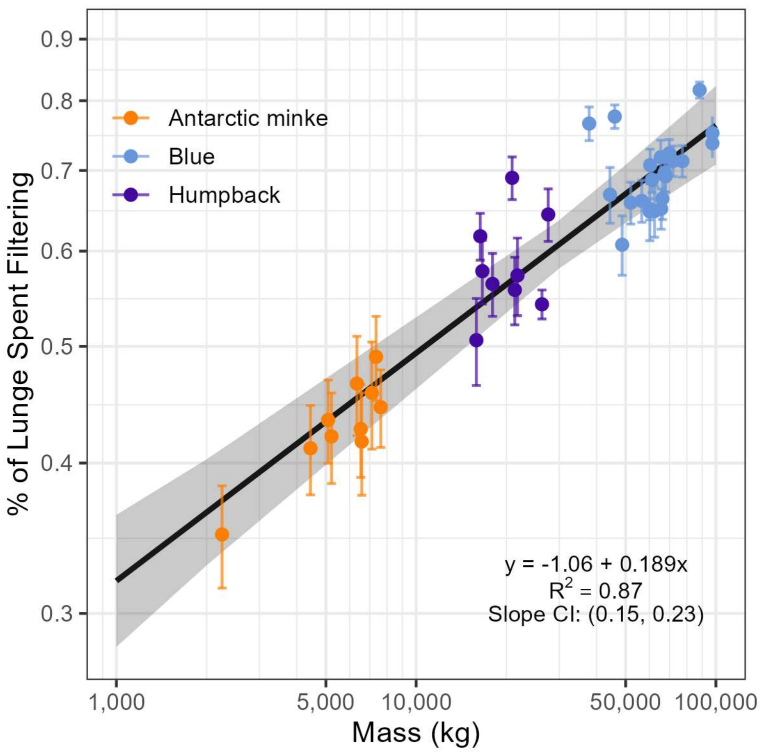


**Figure S1. Allometry of the proportion of time during lunges spent filtering.** Each circle represents one individual tag record and error bars indicate the 95% confidence interval (CI) of the proportion of the lunge cycle spent filtering for all lunges for that individual. Species are indicated by different colors. The solid black line represents the fixed effect model represented by the provided regression equation with the marginal $R^2$ value and CIs (95%) for the regression slope. CIs (95%) are shaded in gray. Data were previously published in Gough et al., 2021 (*115*).

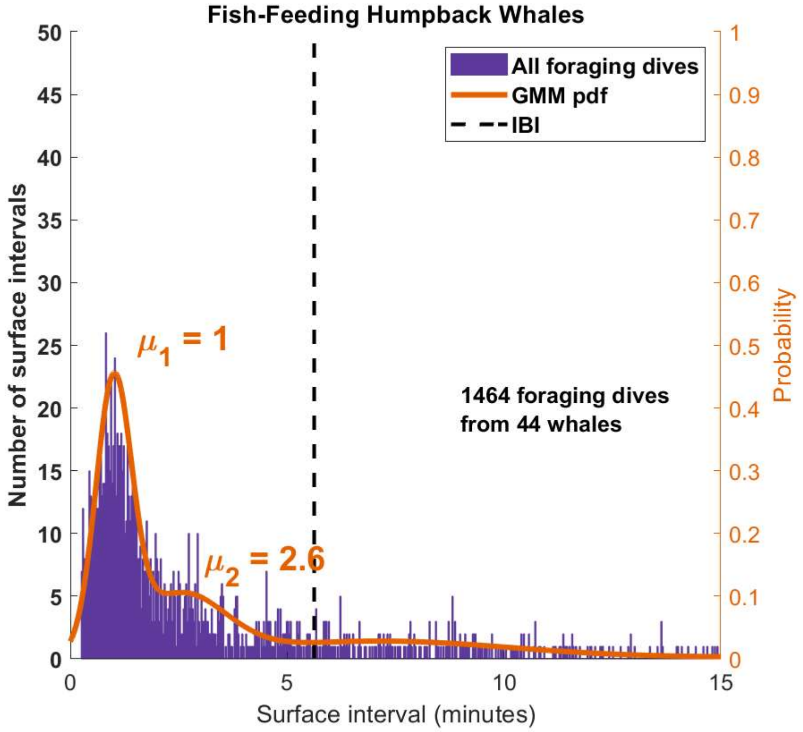


**Figure S2. The distribution of surface intervals following feeding dives of fish-feeding humpback whales.** Purple bars represent counts of surface intervals following feeding dives and the orange line represents the best Gaussian form fit to the distribution. The modes are displayed in orange and the vertical dashed black line represents the threshold for defining dives within a bout, calculated as the final mean + 3 standard deviations as previously used by Cade et al., 2023 and Cade, Seakamela, et al., 2021 (*92*, *100*).

.

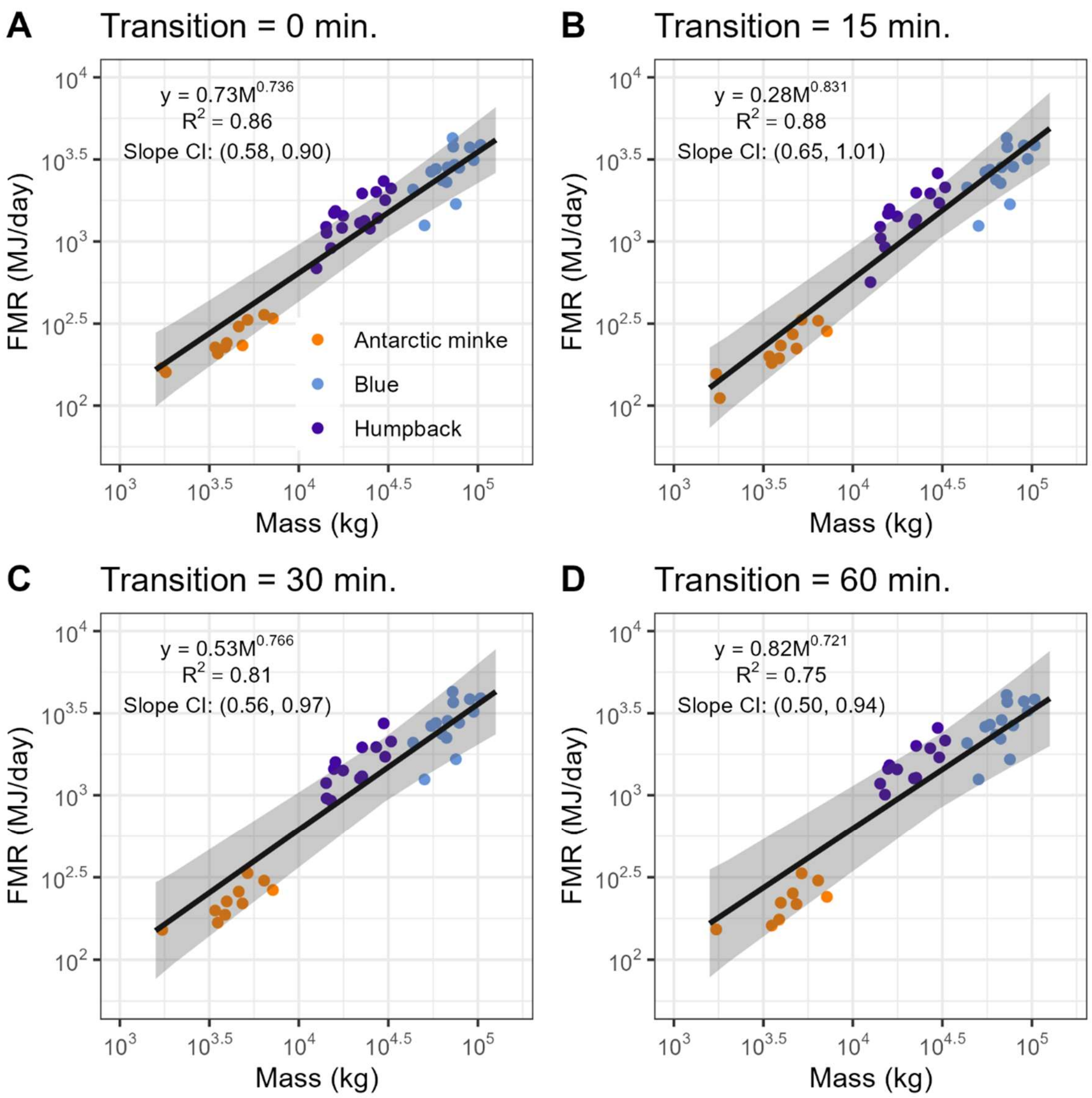


**Figure S3. The allometry of non-foraging FMR with several transition periods dropped from the start of each non-foraging bout.** Each circle represents an individual tag record. Species are indicated by different colors. The solid black line represents the fixed effect model represented by the provided regression equation with the marginal $R^2$ value and CIs (95%) for the regression slope. CIs (95%) are shaded in gray.

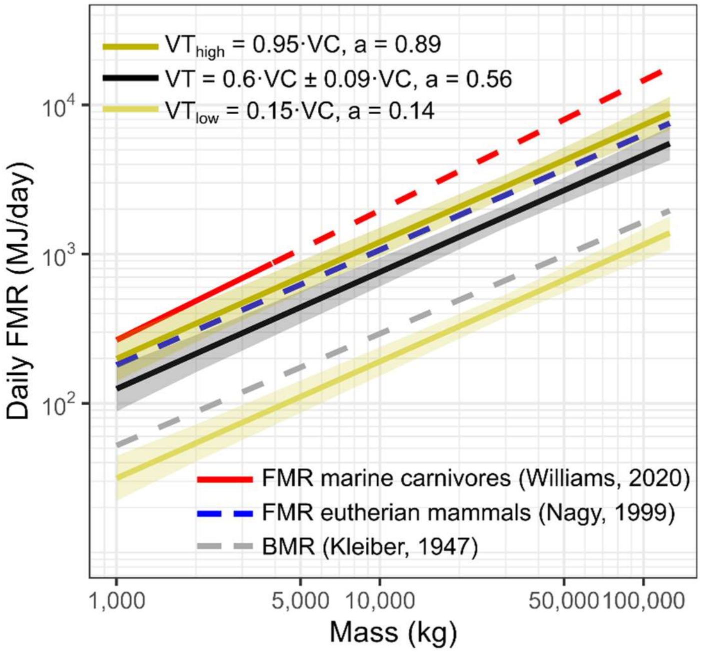


**Figure S4. Sensitivity analysis of the effect of tidal volume ($V_T$) on field metabolic rate (FMR) estimates**. $V_T$s used to inform each FMR scaling relationship are shown in addition to the "a" value representing the intercept of the model (FMR = $aM^b$) for each case. The black line indicates the scenario described as the main result of the paper.

**Table S1. Summary of tag deployments**. Individuals are identified by ID, species code, study area, mass (kg), tidal volume (VT, l), number of breaths, breathing rate ($f_R$, breaths/min), and daily field metabolic rate (FMR, MJ). Species codes are defined as bb = Antarctic minke whale, bw = Blue whale, and mn = Humpback whale.

| ID | Species | Study Area | Mass (kg) | VT (l) | # breaths | $f_R$ (breaths/ min) | FMR (MJ) |
|---|---|---|---|---|---|---|---|
| bb180227-45 | bb | Antarctic | 5173.8 | 179.7 | 2294 | 1.33 | 504.9 |
| bb180304-42 | bb | Antarctic | 6310.0 | 215.6 | 77 | 0.65 | 295.6 |
| bb180304-45 | bb | Antarctic | 7163.3 | 242.6 | 1289 | 0.80 | 410.0 |
| bb180305-42b | bb | Antarctic | 3958.2 | 140.6 | 983 | 1.04 | 309.5 |
| bb190224-48 | bb | Antarctic | 2933.5 | 106.7 | 5 | 1.57 | 354.0 |
| bb190224-52 | bb | Antarctic | 1726.1 | 65.5 | 1954 | 1.55 | 214.2 |
| bb190225-54 | bb | Antarctic | 6203.2 | 212.4 | 415 | 1.09 | 486.6 |
| bb190225-55 | bb | Antarctic | 7652.9 | 257.7 | 402 | 1.04 | 563.7 |
| bb190225-57 | bb | Antarctic | 4980.3 | 173.5 | 420 | 1.22 | 447.7 |
| bb190226-48 | bb | Antarctic | 3525.9 | 126.2 | 1560 | 1.11 | 294.5 |
| bb190226-51 | bb | Antarctic | 4842.2 | 169.2 | 1078 | 0.87 | 312.2 |
| bb190226-53 | bb | Antarctic | 3876.1 | 137.8 | 781 | 1.25 | 363.0 |
| bb190228-52 | bb | Antarctic | 6972.5 | 236.4 | 70 | 0.65 | 323.0 |
| bb190228-55b | bb | Antarctic | 4618.3 | 162.1 | 914 | 1.28 | 436.1 |
| bb190302-48 | bb | Antarctic | 7356.9 | 248.5 | 160 | 1.25 | 653.9 |
| bb190302-52 | bb | Antarctic | 6399.8 | 218.6 | 2973 | 1.16 | 532.7 |
| bb190304-45 | bb | Antarctic | 1806.6 | 68.3 | 673 | 1.21 | 174.3 |
| bb190304-57 | bb | Antarctic | 3409.7 | 122.4 | 650 | 1.06 | 273.0 |
| bw170813-44 | bw | Monterey | 72857.8 | 2048.1 | 2012 | 0.92 | 3972.7 |
| bw170814-31 | bw | Monterey | 68949.5 | 1945.5 | 431 | 1.00 | 4093.6 |
| bw170814-40 | bw | Monterey | 72230.7 | 2032.8 | 1871 | 1.05 | 4514.6 |
| bw170814-50 | bw | Monterey | 74548.3 | 2092.7 | 440 | 1.09 | 4803.9 |
| bw170814-51 | bw | Monterey | 54846.9 | 1578.5 | 1093 | 0.89 | 2957.7 |
| bw170815-21 | bw | Monterey | 72439.3 | 2038.2 | 119 | 1.00 | 4310.4 |
| bw170815-28 | bw | Monterey | 58277.0 | 1668.0 | 1429 | 0.88 | 3089.8 |
| bw170816-27 | bw | Monterey | 72022.6 | 2027.6 | 203 | 0.98 | 4190.8 |
| bw170816-41 | bw | Monterey | 90151.8 | 2489.3 | 743 | 0.87 | 4582.8 |
| bw170816-42 | bw | Monterey | 93972.2 | 2586.0 | 217 | 1.16 | 6322.7 |
| bw170816-44 | bw | Monterey | 64608.3 | 1833.4 | 520 | 1.02 | 3932.1 |
| bw170816-51 | bw | Monterey | 76482.4 | 2143.8 | 166 | 0.80 | 3618.3 |
| bw180827-52 | bw | Monterey | 83871.4 | 2331.4 | 408 | 0.83 | 4078.5 |
| bw180827-53 | bw | Monterey | 75296.4 | 2112.0 | 80 | 0.69 | 3056.9 |
| bw180828-49 | bw | Monterey | 67746.1 | 1915.2 | 841 | 0.70 | 2816.6 |
| bw180829-30 | bw | Monterey | 52055.8 | 1503.7 | 182 | 0.94 | 2972.1 |
| bw180829-47 | bw | Monterey | 78561.3 | 2193.5 | 931 | 0.63 | 2923.8 |
| bw180830-40 | bw | Monterey | 74017.1 | 2079.4 | 374 | 0.76 | 3340.6 |
| bw180830-42 | bw | Monterey | 62982.9 | 1793.4 | 1011 | 0.72 | 2708.0 |
| bw180830-46 | bw | Monterey | 43462.4 | 1273.0 | 342 | 0.83 | 2239.7 |
| bw180830-49 | bw | Monterey | 66853.2 | 1894.3 | 891 | 0.64 | 2561.8 |
| bw180830-52b | bw | Monterey | 66655.8 | 1886.4 | 113 | 0.97 | 3847.5 |

| | | | | | | | |
|---|---|---|---|---|---|---|---|
| bw180904-44 | bw | Monterey | 103676.3 | 2830.6 | 828 | 0.72 | 4289.4 |
| bw180904-48 | bw | Monterey | 75403.7 | 2112.8 | 542 | 0.48 | 2154.5 |
| bw180904-52 | bw | Monterey | 67447.5 | 1907.7 | 22 | 0.66 | 2647.1 |
| bw180905-42 | bw | Monterey | 50331.8 | 1457.0 | 786 | 0.47 | 1446.8 |
| bw180905-53 | bw | Monterey | 94724.4 | 2607.7 | 917 | 0.58 | 3176.8 |
| mn151018-3 | mn | SoCal | 37161.9 | 1102.3 | 133 | 0.94 | 2183.0 |
| mn160421-3 | mn | Monterey | 29785.3 | 898.9 | 134 | 0.45 | 852.8 |
| mn161117-10 | mn | Monterey | 29785.3 | 899.2 | 202 | 0.78 | 1476.1 |
| mn170703-40 | mn | Monterey | 15118.9 | 482.2 | 506 | 0.90 | 911.9 |
| mn170807-40 | mn | SoCal | 16670.1 | 527.6 | 236 | 1.36 | 1514.1 |
| mn170807-41 | mn | SoCal | 26204.7 | 799.3 | 46 | 1.32 | 2221.1 |
| mn170808-42 | mn | SoCal | 8234.6 | 275.6 | 235 | 1.36 | 792.5 |
| mn170809-41 | mn | SoCal | 10069.2 | 331.5 | 147 | 1.17 | 818.4 |
| mn170809-43 | mn | SoCal | 15564.8 | 495.3 | 107 | 1.71 | 1789.2 |
| mn170809-44 | mn | SoCal | 29923.8 | 902.7 | 160 | 1.36 | 2596.6 |
| mn170809-50 | mn | SoCal | 23685.0 | 728.8 | 12 | 1.05 | 1611.3 |
| mn170809-51 | mn | SoCal | 17193.6 | 542.4 | 318 | 0.76 | 873.8 |
| mn170810-30 | mn | SoCal | 46299.6 | 1351.1 | 663 | 1.60 | 4548.6 |
| mn170810-40 | mn | SoCal | 5279.1 | 183.2 | 268 | 1.09 | 421.7 |
| mn170810-42 | mn | SoCal | 13749.0 | 441.7 | 318 | 1.25 | 1166.2 |
| mn170815-20 | mn | Monterey | 20456.0 | 636.1 | 304 | 1.34 | 1805.3 |
| mn170817-30 | mn | Monterey | 23271.6 | 717.3 | 120 | 0.91 | 1378.5 |
| mn170817-43 | mn | Monterey | 24954.6 | 764.7 | 181 | 0.83 | 1331.6 |
| mn170817-50 | mn | Monterey | 32783.4 | 982.3 | 855 | 1.07 | 2208.0 |
| mn170817-51 | mn | Monterey | 17435.1 | 549.6 | 190 | 1.17 | 1351.6 |
| mn180227-40 | mn | Antarctic | 14295.2 | 458.0 | 772 | 1.49 | 1441.3 |
| mn180228-47 | mn | Antarctic | 12541.7 | 406.0 | 1867 | 1.54 | 1318.2 |
| mn180302-27 | mn | Antarctic | 22573.9 | 697.0 | 1548 | 1.35 | 1987.5 |
| mn180302-47 | mn | Antarctic | 22573.9 | 697.1 | 1762 | 1.25 | 1837.9 |
| mn180831-11 | mn | Monterey | 31332.0 | 941.3 | 338 | 1.19 | 2363.4 |
| mn180831-20 | mn | Monterey | 27561.2 | 837.1 | 123 | 0.87 | 1538.5 |
| mn180831-30 | mn | Monterey | 7107.8 | 240.4 | 278 | 0.95 | 480.0 |
| mn180831-44 | mn | Monterey | 32562.9 | 976.9 | 60 | 0.94 | 1942.5 |
| mn180906-40 | mn | Monterey | 14638.3 | 468.1 | 132 | 1.01 | 996.6 |
| mn181002-45 | mn | Monterey | 27039.1 | 823.4 | 1301 | 1.23 | 2137.9 |
| mn181002-53 | mn | Monterey | 27039.1 | 823.0 | 68 | 0.98 | 1701.6 |
| mn181003-53 | mn | Monterey | 25139.5 | 769.0 | 314 | 1.42 | 2299.8 |
| mn190228-42 | mn | Antarctic | 16019.3 | 509.1 | 3487 | 1.38 | 1476.7 |
| mn190228-44 | mn | Antarctic | 21946.6 | 678.8 | 1061 | 1.06 | 1525.1 |
| mn190305-44 | mn | Antarctic | 15700.2 | 499.5 | 3244 | 1.69 | 1781.0 |
| mn190306-42 | mn | Antarctic | 30341.8 | 914.1 | 1094 | 1.04 | 2015.5 |
| mn190306-45 | mn | Antarctic | 14210.3 | 455.5 | 868 | 1.66 | 1591.1 |
| mn190309-44b | mn | Antarctic | 17678.9 | 556.6 | 1792 | 1.38 | 1621.8 |

**Table S2. Mean ± S.D. of species body size and tag data parameters for foraging and non-foraging bouts.**

| | ***B. bonaerensis*** | ***M. novaeangliae*** | ***B. musculus*** |
|---|---|---|---|
| Body Mass (kg) | 4,227 ± 1,680 | 21,435 ± 6,324 | 71,075 ± 16,450 |
| Foraging Bout $f_R$ (breaths $min^{-1}$) | 1.32 ± 0.27 | 1.33 ± 0.34 | 0.81 ± 0.15 |
| Non-Foraging Bout $f_R$ (breaths $min^{-1}$) | 0.86 ± 0.18 | 1.06 ± 0.22 | 0.67 ± 0.16 |
| Foraging Bout Speed (m $s^{-1}$) | 1.79 ± 0.20 | 1.43 ± 0.16 | 1.50 ± 0.20 |
| Non-Foraging Bout Speed (m $s^{-1}$) | 1.91 ± 0.17 | 1.64 ± 0.23 | 1.74 ± 0.34 |
| Foraging Bout Angular Velocity (° $min^{-1}$) | 190.8 ± 31.2 | 145.8 ± 30.2 | 87.9 ± 27.6 |
| Non-Foraging Bout Angular Velocity (° $min^{-1}$) | 86.3 ± 22.2 | 80.0 ± 62.4 | 61.6 ± 18.0 |
| Total Foraging Hours (n = # of individuals) | 140.4 (11) | 155.4 (18) | 163.9 (15) |
| Total Non-Foraging Hours (n = # of individuals) | 80.6 (11) | 135.9 (18) | 162.4 (15) |

## REFERENCES AND NOTES


1. M. Kleiber, Body size and metabolism. *Hilgardia* **6**, 316–352 (1932).
2. M. Kleiber, *The Fire of Life. An Introduction to Animal Energetics*. (1961).
3. R. H. Peters, *The Ecological Implications of Body Size* (Cambridge Univ. Press, 1983) *Cambridge Studies in Ecology*.
4. K. Schmidt-Nielsen, *Scaling: Why Is Animal Size so Important?* (Cambridge Univ. Press, 1984).
5. J. H. Brown, J. R. Burger, C. Hou, C. A. S. Hall, The pace of life: Metabolic energy, biological time, and life history. *Integr. Comp. Biol.,* icac058 (2022).
6. D. S. Glazier, Beyond the "3/4-power law": Variation in the intra- and interspecific scaling of metabolic rate in animals. *Biol. Rev. Camb. Philos. Soc.* **80**, 611–662 (2005).
7. D. S. Glazier, Variable metabolic scaling breaks the law: From 'Newtonian' to 'Darwinian' approaches. *Proc. Biol. Sci.* **289**, 20221605 (2022).
8. C. R. White, P. Cassey, T. M. Blackburn, Allometric exponents do not support a universal metabolic allometry. *Ecology* **88**, 315–323 (2007).
9. C. R. White, L. A. Alton, C. L. Bywater, E. J. Lombardi, D. J. Marshall, Metabolic scaling is the product of life-history optimization. *Science* **377**, 834–839 (2022).
10. J. Weiner, Physiological limits to sustainable energy budgets in birds and mammals: Ecological implications. *Trends Ecol. Evol.* **7**, 384–388 (1992).
11. J. A. Goldbogen, Physiological constraints on marine mammal body size. *Proc. Natl. Acad. Sci. U.S.A.* **115**, 3995–3997 (2018).
12. M. S. Savoca, M. F. Czapanskiy, S. R. Kahane-Rapport, W. T. Gough, J. A. Fahlbusch, K. C. Bierlich, P. S. Segre, J. Di Clemente, G. S. Penry, D. N. Wiley, J. Calambokidis, D. P. Nowacek,

D. W. Johnston, N. D. Pyenson, A. S. Friedlaender, E. L. Hazen, J. A. Goldbogen, Baleen whale prey consumption based on high-resolution foraging measurements. *Nature* **599**, 85–90 (2021).

13. T. M. Williams, J. Haun, R. W. Davis, L. A. Fuiman, S. Kohin, A killer appetite: Metabolic consequences of carnivory in marine mammals. *Comp. Biochem. Physiol. A Mol. Integr. Physiol.* **129**, 785–796 (2001).

14. T. M. Williams, M. Peter-Heide Jørgensen, A. M. Pagano, C. M. Bryce, Hunters versus hunted: New perspectives on the energetic costs of survival at the top of the food chain. *Funct. Ecol.* **34**, 2015–2029 (2020).

15. S. K. A. Videsen, M. Simon, F. Christiansen, A. Friedlaender, J. Goldbogen, H. Malte, P. Segre, T. Wang, M. Johnson, P. T. Madsen, Cheap gulp foraging of a giga-predator enables efficient exploitation of sparse prey. *Sci. Adv.* **9**, eade3889 (2023).

16. K. C. Bierlich, J. Hewitt, R. S. Schick, L. Pallin, J. Dale, A. S. Friedlaender, F. Christiansen, K. R. Sprogis, A. H. Dawn, C. N. Bird, G. D. Larsen, R. Nichols, M. R. Shero, J. Goldbogen, A. J. Read, D. W. Johnston, Seasonal gain in body condition of foraging humpback whales along the Western Antarctic Peninsula. *Front. Mar. Sci.* **9**, doi.org/10.3389/fmars.2022.1036860 (2022).

17. H. Omura, S. Ohsumi, T. Nemoto, K. Nasu, T. Kasuya, Black right whales in the North Pacific. *Sci. Rep. Whales Res. Inst.*, 1–78 (1969).

18. L. Riekkola, V. Andrews-Goff, A. Friedlaender, A. N. Zerbini, R. Constantine, Longer migration not necessarily the costliest strategy for migrating humpback whales. *Aquat. Conserv. Mar. Freshw. Ecosyst.* **30**, 937–948 (2020).

19. F. Christiansen, M. M. Uhart, L. Bejder, P. Clapham, Y. Ivashchenko, D. Tormosov, N. Lewin, M. Sironi, Fetal growth, birth size and energetic cost of gestation in southern right whales. *J. Physiol.* **600**, 2245–2266 (2022).

20. F. Christiansen, K. R. Sprogis, M. L. K. Nielsen, M. Glarou, L. Bejder, Energy expenditure of southern right whales varies with body size, reproductive state and activity level. *J. Exp. Biol.* **226**, jeb245137 (2023).

21. S. Villegas-Amtmann, L. K. Schwarz, J. L. Sumich, D. P. Costa, A bioenergetics model to evaluate demographic consequences of disturbance in marine mammals applied to gray whales. *Ecosphere* **6**, art183 (2015).

22. F. Christiansen, P. T. Madsen, V. Andrews-Goff, M. C. Double, J. R. How, P. Clapham, Y. Ivashchenko, D. Tormosov, K. R. Sprogis, Extreme capital breeding for giants: Effects of body size on humpback whale energy expenditure and fasting endurance. *Ecol. Model.* **501**, 110994 (2025).

23. M. Simon, M. Johnson, P. Tyack, P. T. Madsen, Behaviour and kinematics of continuous ram filtration in bowhead whales (Balaena mysticetus). *Proc. Biol. Sci.* **276**, 3819–3828 (2009).

24. J. M. van der Hoop, A. E. Nousek-McGregor, D. P. Nowacek, S. E. Parks, P. Tyack, P. T. Madsen, Foraging rates of ram-filtering North Atlantic right whales. *Funct. Ecol.* **33**, 1290–1306 (2019).

25. S. R. Kahane-Rapport, M. S. Savoca, D. E. Cade, P. S. Segre, K. C. Bierlich, J. Calambokidis, J. Dale, J. A. Fahlbusch, A. S. Friedlaender, D. W. Johnston, A. J. Werth, J. A. Goldbogen, Lunge filter feeding biomechanics constrain rorqual foraging ecology across scale. *J. Exp. Biol.* **223**, jeb224196 (2020).

26. M. Simon, M. Johnson, P. T. Madsen, Keeping momentum with a mouthful of water: Behavior and kinematics of humpback whale lunge feeding. *J. Exp. Biol.* **215**, 3786–3798 (2012).

27. D. E. Cade, A. S. Friedlaender, J. Calambokidis, J. A. Goldbogen, Kinematic diversity in rorqual whale feeding mechanisms. *Curr. Biol.* **26**, 2617–2624 (2016).

28. A. Acevedo-Gutiérrez, D. A. Croll, B. R. Tershy, High feeding costs limit dive time in the largest whales. *J. Exp. Biol.* **205**, 1747–1753 (2002).

29. J. A. Goldbogen, N. D. Pyenson, R. E. Shadwick, Big gulps require high drag for fin whale lunge feeding. *Mar. Ecol. Prog. Ser.* **349**, 289–301 (2007).

30. W. T. Gough, D. E. Cade, M. F. Czapanskiy, J. Potvin, F. E. Fish, S. R. Kahane-Rapport, M. S. Savoca, K. C. Bierlich, D. W. Johnston, A. S. Friedlaender, A. Szabo, L. Bejder, J. A. Goldbogen, Fast and furious: Energetic trade-offs and scaling of high-speed foraging in rorqual whales. *Integr. Org. Biol.* **4**, obac038 (2022).

31. A. S. Blix, L. P. Folkow, Daily energy expenditure in free living minke whales. *Acta Physiol. Scand.* **153**, 61–66 (1995).

32. J. L. Sumich, Swimming velocities, breathing patterns, and estimated costs of locomotion in migrating gray whales, *Eschrichtius robustus*. *Can. J. Zool.* **61**, 647–652 (1983).

33. A. Fahlman, J. van der Hoop, M. J. Moore, G. Levine, J. Rocho-Levine, M. Brodsky, Estimating energetics in cetaceans from respiratory frequency: Why we need to understand physiology. *Biol. Open* **5**, 436–442 (2016).

34. J. A. Goldbogen, J. Calambokidis, D. A. Croll, M. F. McKenna, E. Oleson, J. Potvin, N. D. Pyenson, G. Schorr, R. E. Shadwick, B. R. Tershy, Scaling of lunge-feeding performance in rorqual whales: Mass-specific energy expenditure increases with body size and progressively limits diving capacity. *Funct. Ecol.* **26**, 216–226 (2012).

35. T. M. Williams, R. W. Davis, L. A. Fuiman, J. Francis, B. J. Le, Boeuf, M. Horning, J. Calambokidis, D. A. Croll, Sink or swim: Strategies for cost-efficient diving by marine mammals. *Science* **288**, 133–136 (2000).

36. J. A. Goldbogen, J. Calambokidis, R. E. Shadwick, E. M. Oleson, M. A. McDonald, J. A. Hildebrand, Kinematics of foraging dives and lunge-feeding in fin whales. *J. Exp. Biol.* **209**, 1231–1244 (2006).

37. J. A. Goldbogen, J. Calambokidis, A. S. Friedlaender, J. Francis, S. L. DeRuiter, A. K. Stimpert, E. Falcone, B. L. Southall, Underwater acrobatics by the world's largest predator: 360° rolling manoeuvres by lunge-feeding blue whales. *Biol. Lett.* **9**, 20120986 (2013).

38. R. P. Wilson, I. W. Griffiths, P. A. Legg, M. I. Friswell, O. R. Bidder, L. G. Halsey, S. A. Lambertucci, E. L. C. Shepard, Turn costs change the value of animal search paths. *Ecol. Lett.* **16**, 1145–1150 (2013).

39. R. P. Wilson, K. A. R. Rose, R. S. Metcalfe, M. D. Holton, J. Redcliffe, R. Gunner, L. Börger, A. Loison, M. Jezek, M. S. Painter, V. Silovský, N. Marks, M. Garel, C. Toïgo, P. Marchand, N. C. Bennett, M. A. McNarry, K. A. Mackintosh, M. R. Brown, D. M. Scantlebury, Path tortuosity changes the transport cost paradigm in terrestrial animals. *Ecography* **44**, 1524–1532 (2021).

40. W. Gearty, C. R. McClain, J. L. Payne, Energetic tradeoffs control the size distribution of aquatic mammals. *Proc. Natl. Acad. Sci. U.S.A.* **115**, 4194–4199 (2018).

41. J. A. Goldbogen, D. E. Cade, D. M. Wisniewska, J. Potvin, P. S. Segre, M. S. Savoca, E. L. Hazen, M. F. Czapanskiy, S. R. Kahane-Rapport, S. L. DeRuiter, S. Gero, P. Tønnesen, W. T. Gough, M. B. Hanson, M. M. Holt, F. H. Jensen, M. Simon, A. K. Stimpert, P. Arranz, D. W. Johnston, D. P. Nowacek, S. E. Parks, F. Visser, A. S. Friedlaender, P. L. Tyack, P. T. Madsen, N. D. Pyenson, Why whales are big but not bigger: Physiological drivers and ecological limits in the age of ocean giants. *Science* **366**, 1367–1372 (2019).

42. J. J. Videler, D. Weihs, Energetic advantages of burst-and-coast swimming of fish at high speeds. *J. Exp. Biol.* **97**, 169–178 (1982).

43. D. P. Nowacek, M. P. Johnson, P. L. Tyack, K. A. Shorter, W. A. McLellan, D. A. Pabst, Buoyant balaenids: The ups and downs of buoyancy in right whales. *Proc. Biol. Sci.* **268**, 1811–1816 (2001).

44. D. Weihs, Energetic advantages of burst swimming of fish. *J. Theor. Biol.* **48**, 215–229 (1974).

45. T. M. Williams, Intermittent swimming by mammals: A strategy for increasing energetic efficiency during diving1. *Am. Zool.* **41**, 166–176 (2001).

46. D. A. S. Rosen, A. J. Winship, L. A. Hoopes, Thermal and digestive constraints to foraging behaviour in marine mammals. *Philos. Trans. R Soc. Lond. B Biol. Sci.* **362**, 2151–2168 (2007).

47. C. G. Booth, M. Guilpin, A.-K. Darias-O'Hara, J. M. Ransijn, M. Ryder, D. Rosen, E. Pirotta, S. Smout, E. A. Mc Huron, J. Nabe-Nielsen, D. P. Costa, Estimating energetic intake for marine mammal bioenergetic models. *Conserv. Physiol.* **11**, coac083 (2023).

48. D. A. S. Rosen, A. W. Trites, Heat increment of feeding in Steller sea lions, *Eumetopias jubatus*. *Comp. Biochem. Physiol. A Physiol.* **118**, 877–881 (1997).

49. L. C. Yeates, D. S. Houser, Thermal tolerance in bottlenose dolphins (Tursiops truncatus). *J. Exp. Biol.* **211**, 3249–3257 (2008).

50. R. W. Davis, M. A. Castellini, G. L. Kooyman, R. Maue, Renal glomerular filtration rate and hepatic blood flow during voluntary diving in Weddell seals. *Am. J. Physiol.* **245**, R743–R748 (1983).

51. J. A. Goldbogen, D. E. Cade, J. Calambokidis, M. F. Czapanskiy, J. Fahlbusch, A. S. Friedlaender, W. T. Gough, S. R. Kahane-Rapport, M. S. Savoca, K. V. Ponganis, P. J. Ponganis, Extreme bradycardia and tachycardia in the world's largest animal. *Proc. Natl. Acad. Sci. U.S.A.* **116**, 25329–25332 (2019).

52. S. W. Grinnell, L. Irving, P. F. Scholander, Experiments on the relation between blood flow and heart rate in the diving seal. *J. Cell. Comp. Physiol.* **19**, 341–350 (1942).

53. W. M. Zapol, G. C. Liggins, R. C. Schneider, J. Qvist, M. T. Snider, R. K. Creasy, P. W. Hochachka, Regional blood flow during simulated diving in the conscious Weddell seal. *J. Appl. Physiol. Respir. Environ. Exerc. Physiol.* **47**, 968–973 (1979).

54. D. A. S. Rosen, C. D. Gerlinsky, A. W. Trites, Evidence of partial deferment of digestion during diving in Steller sea lions (*Eumetopias jubatus*). *J. Exp. Mar. Biol. Ecol.* **469**, 93–97 (2015).

55. D. G. Ainley, R. P. Wilson, "Hot penguins: Cold water" in *The Aquatic World of Penguins* (Springer, 2023), pp. 217–256; https://link.springer.com/chapter/10.1007/978-3-031-33990-5_7.

56. J. E. Heyning, J. G. Mead, Thermoregulation in the mouths of feeding gray whales. *Science* **278**, 1138–1139 (1997).

57. E. G. Ekdale, S. S. Kienle, Passive restriction of blood flow and counter-current heat exchange via lingual retia in the tongue of a neonatal gray whale *Schrichtius robustus* (*Cetacea*, *Mysticeti*). *Anat. Rec.* **298**, 675–679 (2015).

58. A. J. Werth, Adaptations of the cetacean hyolingual apparatus for aquatic feeding and thermoregulation. *Anat. Rec.* **290**, 546–568 (2007).

59. J. E. Heyning, J. G. Mead, M. M. Bryden, A palatal rete in the right whale? *Nature* **361**, 24–25 (1993).

60. T. J. Ford, S. D. Kraus, A rete in the right whale. *Nature* **359**, 680–680 (1992).

61. R. P. Wilson, B. M. Culik, The cost of a hot meal: Facultative specific dynamic action may ensure temperature homeostasis in post-ingestive endotherms. *Comp. Biochem. Physiol. A Physiol.* **100**, 151–154 (1991).

62. M. Glarou, M. H. Rasmussen, A. Poldner, S. N. S. Ruppert, D. Sotiropoulou, Z. Sadozai, M. Jarzynowska, M. R. Iversen, G. M. Sigurðsson, S. D. Halldórsson, V. Chosson, P. J. Clapham, Y. Ivashchenko, D. Tormosov, F. Christiansen, Size-specific strategies of sympatric cetaceans to reduce heat loss. *J. Exp. Biol.* **228**, jeb249356 (2025).

63. A. B. Favilla, D. P. Costa, Thermoregulatory strategies of diving air-breathing marine vertebrates: A review. *Front. Ecol. Evol.* **8** (2020).

64. K. A. Nagy, I. A. Girard, T. K. Brown, Energetics of free-ranging mammals, reptiles, and birds. *Annu. Rev. Nutr.* **19**, 247–277 (1999).

65. J. A. Goldbogen, E. L. Hazen, A. S. Friedlaender, J. Calambokidis, S. L. DeRuiter, A. K. Stimpert, B. L. Southall, Prey density and distribution drive the three-dimensional foraging strategies of the largest filter feeder. *Funct. Ecol.* **29**, 951–961 (2015).

66. E. L. Hazen, A. S. Friedlaender, J. A. Goldbogen, Blue whales (*Balaenoptera musculus*) optimize foraging efficiency by balancing oxygen use and energy gain as a function of prey density. *Sci. Adv.* **1**, e1500469 (2015).

67. D. Croll, B. Marinovic, S. Benson, F. Chavez, N. Black, R. Ternullo, B. Tershy, From wind to whales: Trophic links in a coastal upwelling system. *Mar. Ecol. Prog. Ser.* **289**, 117–130 (2005).

68. Y. Akiyama, T. Akamatsu, M. H. Rasmussen, M. R. Iversen, T. Iwata, Y. Goto, K. Aoki, K. Sato, Leave or stay? Video-logger revealed foraging efficiency of humpback whales under temporal change in prey density. *PLOS ONE* **14**, e0211138 (2019).

69. J. A. Goldbogen, N. D. Pyenson, P. T. Madsen, How whales dive, feast, and fast: The ecophysiological drivers and limits of foraging in the evolution of cetaceans. *Annu. Rev. Ecol. Evol. Syst.* **54**, 307–325 (2023).

70. G. J. Slater, J. A. Goldbogen, N. D. Pyenson, Independent evolution of baleen whale gigantism linked to Plio-Pleistocene ocean dynamics. *Proc. Biol. Sci.* **284**, 20170546 (2017).

71. E. Pirotta, M. Mangel, D. P. Costa, B. Mate, J. A. Goldbogen, D. M. Palacios, L. A. Hückstädt, E. A. McHuron, L. Schwarz, L. New, A dynamic state model of migratory behavior and physiology to assess the consequences of environmental variation and anthropogenic disturbance on marine vertebrates. *Am. Nat.* **191**, E40–E56 (2018).

72. B. Abrahms, E. L. Hazen, E. O. Aikens, M. S. Savoca, J. A. Goldbogen, S. J. Bograd, M. G. Jacox, L. M. Irvine, D. M. Palacios, B. R. Mate, Memory and resource tracking drive blue whale migrations. *Proc. Natl. Acad. Sci. U.S.A.* **116**, 5582–5587 (2019).

73. J. A. Fahlbusch, M. F. Czapanskiy, J. Calambokidis, D. E. Cade, B. Abrahms, E. L. Hazen, J. A. Goldbogen, Blue whales increase feeding rates at fine-scale ocean features. *Proc. Biol. Sci.* **289**, 20221180 (2022).

74. C. Lockyer, Body weights of some species of large whales. *IJMS* **36**, 259–273 (1976).

75. A. R. Evans, D. Jones, A. G. Boyer, J. H. Brown, D. P. Costa, S. K. M. Ernest, E. M. G. Fitzgerald, M. Fortelius, J. L. Gittleman, M. J. Hamilton, L. E. Harding, K. Lintulaakso, S. K. Lyons, J. G. Okie, J. J. Saarinen, R. M. Sibly, F. A. Smith, P. R. Stephens, J. M. Theodor, M. D. Uhen, The maximum rate of mammal evolution. *Proc. Natl. Acad. Sci. U.S.A.* **109**, 4187–4190 (2012).

76. J. L. Maresh, "Bioenergetics of Marine Mammals: The Influence of Body Size, Reproductive Status, Locomotion and Phylogeny on Metabolism," thesis, UC Santa Cruz, Santa Cruz, CA. (2014).

77. N. Bose, J. Lien, J. Ahia, Measurements of the bodies and flukes of several cetacean species. *Proc. Biol. Sci.* **242**, 163–173 (1997).

78. H. Pontzer, D. A. Raichlen, R. W. Shumaker, C. Ocobock, S. A. Wich, Metabolic adaptation for low energy throughput in orangutans. *Proc. Natl. Acad. Sci. U.S.A.* **107**, 14048–14052 (2010).

79. K. Healy, T. H. G. Ezard, O. R. Jones, R. Salguero-Gómez, Y. M. Buckley, Animal life history is shaped by the pace of life and the distribution of age-specific mortality and reproduction. *Nat. Ecol. Evol.* **3**, 1217–1224 (2019).

80. J. M. Jeschke, H. Kokko, The roles of body size and phylogeny in fast and slow life histories. *Evol. Ecol.* **23**, 867–878 (2009).

81. S. C. Stearns, The influence of size and phylogeny on patterns of covariation among life-history traits in the mammals. *Oikos* **41**, 173–187 (1983).

82. C. Lockyer, Review of baleen whale (*Mysticeti*) reproduction and implications for management. *IWC*, **6**, 27–50 (1984).

83. L. Bejder, S. Videsen, L. Hermannsen, M. Simon, D. Hanf, P. T. Madsen, Low energy expenditure and resting behaviour of humpback whale mother-calf pairs highlights conservation importance of sheltered breeding areas. *Sci. Rep.* **9**, 771 (2019).

84. C. J. Downs, J. L. Brown, B. Wone, E. R. Donovan, K. Hunter, J. P. Hayes, Selection for increased mass-independent maximal metabolic rate suppresses innate but not adaptive immune function. *Proc. Biol. Sci.* **280**, 20122636 (2013).

85. A. C. Steyermark, A high standard metabolic rate constrains juvenile growth. *Zoology* **105**, 147–151 (2002).

86. K. R. Westerterp, Control of energy expenditure in humans. *Eur. J. Clin. Nutr.* **71**, 340–344 (2017).

87. H. Pontzer, R. Durazo-Arvizu, L. Dugas, J. Plange-Rhule, P. Bovet, T. E. Forrester, E. V. Lambert, R. S. Cooper, D. A. Schoeller, A. Luke, Constrained total energy expenditure and metabolic adaptation to physical activity in adult humans. *Curr. Biol.* **26**, 410–417 (2016).

88. B. R. Mate, V. Yu. Ilyashenko, A. L. Bradford, V. V. Vertyankin, G. A. Tsidulko, V. V. Rozhnov, L. M. Irvine, Critically endangered western gray whales migrate to the eastern North Pacific. *Biol. Lett.* **11**, 20150071 (2015).

89. D. J. McCauley, The future of whales in our Anthropocene ocean. *Sci. Adv.s* **9**, eadi7604 (2023).

90. N. S. Diffenbaugh, C. B. Field, Changes in ecologically critical terrestrial climate conditions. *Science* **341**, 486–492 (2013).

91. E. S. Poloczanska, C. J. Brown, W. J. Sydeman, W. Kiessling, D. S. Schoeman, P. J. Moore, K. Brander, J. F. Bruno, L. B. Buckley, M. T. Burrows, C. M. Duarte, B. S. Halpern, J. Holding, C. V. Kappel, M. I. O'Connor, J. M. Pandolfi, C. Parmesan, F. Schwing, S. A. Thompson, A. J. Richardson, Global imprint of climate change on marine life. *Nat. Clim. Chang.* **3**, 919–925 (2013).

92. D. E. Cade, S. R. Kahane-Rapport, W. T. Gough, K. C. Bierlich, J. M. J. Linsky, J. Calambokidis, D. W. Johnston, J. A. Goldbogen, A. S. Friedlaender, Minke whale feeding rate limitations suggest constraints on the minimum body size for engulfment filtration feeding. *Nat. Ecol. Evol.* **7**, 535–546 (2023).

93. P. S. Segre, W. T. Gough, E. A. Roualdes, D. E. Cade, M. F. Czapanskiy, J. Fahlbusch, S. R. Kahane-Rapport, W. K. Oestreich, L. Bejder, K. C. Bierlich, J. A. Burrows, J. Calambokidis, E. M. Chenoweth, J. di Clemente, J. W. Durban, H. Fearnbach, F. E. Fish, A. S. Friedlaender, P. Hegelund, D. W. Johnston, D. P. Nowacek, M. G. Oudejans, G. S. Penry, J. Potvin, M. Simon, A. Stanworth, J. M. Straley, A. Szabo, S. K. A. Videsen, F. Visser, C. R. Weir, D. N. Wiley, J. A. Goldbogen, Scaling of maneuvering performance in baleen whales: Larger whales outperform expectations. *J. Exp. Biol.* **225**, jeb243224 (2022).

94. D. E. Cade, W. T. Gough, M. F. Czapanskiy, J. A. Fahlbusch, S. R. Kahane-Rapport, J. M. J. Linsky, R. C. Nichols, W. K. Oestreich, D. M. Wisniewska, A. S. Friedlaender, J. A. Goldbogen, Tools for integrating inertial sensor data with video bio-loggers, including estimation of animal orientation, motion, and position. *Anim. Biotelemetry* **9**, 34 (2021).

95. D. E. Cade, K. R. Barr, J. Calambokidis, A. S. Friedlaender, J. A. Goldbogen, Determining forward speed from accelerometer jiggle in aquatic environments. *J. Exp. Biol.* **221**, jeb170449 (2018).

96. J. D. Burnett, L. Lemos, D. Barlow, M. G. Wing, T. Chandler, L. G. Torres, Estimating morphometric attributes of baleen whales with photogrammetry from small UASs: A case study with blue and gray whales. *Mar. Mamm. Sci.* **35**, 108–139 (2019).

97. W. I. Torres, K. Bierlich, MorphoMetriX: A photogrammetric measurement GUI for morphometric analysis of megafauna. *J. Open Source Softw.* **5**, 1825 (2020).

98. J. A. Goldbogen, J. Calambokidis, D. A. Croll, J. T. Harvey, K. M. Newton, E. M. Oleson, G. Schorr, R. E. Shadwick, Foraging behavior of humpback whales: Kinematic and respiratory patterns suggest a high cost for a lunge. *J. Exp. Biol.* **211**, 3712–3719 (2008).

99. A. M. Blawas, respdetect: A tool to detect respirations from kinematic data (2025). https://github.com/ashleyblawas/respdetect.

100. D. E. Cade, S. M. Seakamela, K. P. Findlay, J. Fukunaga, S. R. Kahane-Rapport, J. D. Warren, J. Calambokidis, J. A. Fahlbusch, A. S. Friedlaender, E. L. Hazen, D. Kotze, S. McCue,

M. Meÿer, W. K. Oestreich, M. G. Oudejans, C. Wilke, J. A. Goldbogen, Predator-scale spatial analysis of intra-patch prey distribution reveals the energetic drivers of rorqual whale super-group formation. *Funct. Ecol.* **35**, 894–908 (2021).

101. G. L. Kooyman, Respiratory adaptations in marine mammals. *Am. Zool.* **13**, 457–468 (1973).

102. C. R. Olsen, F. C. Hale, R. Elsner, Mechanics of ventilation in the pilot whale. *Respir. Physiol.* **7**, 137–149 (1969).

103. A. Fahlman, S. H. Loring, G. Levine, J. Rocho-Levine, T. Austin, M. Brodsky, Lung mechanics and pulmonary function testing in cetaceans. *J. Exp. Biol.* **218**, 2030–2038 (2015).

104. G. L. Kooyman, K. S. Norris, R. L. Gentry, Spout of the gray whale: Its physical characteristics. *Science* **190**, 908–910 (1975).

105. G. L. Kooyman, L. H. Cornell, Flow properties of expiration and inspiration in a trained bottle-mosed porpoise. *Physiol. Zool.* **54**, 55–61 (1981).

106. L. Irving, P. F. Scholander, S. W. Grinnell, The respiration of the porpoise, tursiops truncatus. *J. Cell. Comp. Physiol.* **17**, 145–168 (1941).

107. B. Kriete, "Bioenergetics in the Killer Whale, O*rcinus orca*," thesis, University of British Columbia, Vancouver, Canada (1994).

108. N. W. Kasting, S. A. L. Adderley, T. Safford, K. G. Hewlett, Thermoregulation in beluga (*Delphinapterus leucas*) and killer (*Orcinus orca*) whales. *Physiol. Zool.* **62**, 687–701 (1989).

109. E. A. Wahrenbrock, E. I. Eger, R. B. Laravuso, G. Maruschak, Anesthetic uptake—Of mice and men (and whales). *Anesthesiology* **40**, 19–23 (1974).

110. J. P. Mortola, J. Seguin, End-tidal $CO_2$ in some aquatic mammals of large size. *Zoology (Jena)* **112**, 77–85 (2009).

111. R Core Team, R: A Language and Environment for Statistical Computing, R Foundation for Statistical Computing (2020); https://R-project.org.

112. S. Nakagawa, H. Schielzeth, A general and simple method for obtaining R2 from generalized linear mixed-effects models. *Methods Ecol. Evol.* **4**, 133–142 (2013).

113. S. R. Kahane-Rapport, J. A. Goldbogen, Allometric scaling of morphology and engulfment capacity in rorqual whales. *J. Morphol.* **279**, 1256–1268 (2018).

114. M. Kleiber, Metabolic turnover rate: A physiological meaning of the metabolic rate per unit body weight. *J. Theor. Biol.* **53**, 199–204 (1975).

115. W. T. Gough, H. J. Smith, M. S. Savoca, M. F. Czapanskiy, F. E. Fish, J. Potvin, K. C. Bierlich, D. E. Cade, J. Di Clemente, J. Kennedy, P. Segre, A. Stanworth, C. Weir, J. A. Goldbogen, Scaling of oscillatory kinematics and Froude efficiency in baleen whales. *J. Exp. Biol.* **224**, jeb237586 (2021).
